# A simple mechanism for higher-order correlations in integrate-and-fire neurons

**DOI:** 10.1186/1471-2202-13-S1-P45

**Published:** 2012-07-16

**Authors:** David A Leen, Eric Shea-Brown

**Affiliations:** 1 Department of Applied Mathematics, University of Washington, Seattle, WA 98195, USA; 2 Program in Neurobiology and Behavior, University of Washington, Seattle, WA, 98195, USA

## 

Recent work [1] shows that common input gives rise to higher-order correlations in the Dichotomized Gaussian neuron model. Here we study a homogeneous population of integrate-and-fire neurons receiving correlated input. Each neuron receives an independent white noise input and all neurons receive a common Gaussian input. To quantify the contributions of higher-order correlations we use a maximum entropy model. The model with interactions up to second order (i.e. pairwise correlations) is known as the Ising model. The Kullbach-Leibler divergence between the Ising model and the model with interactions of all orders allows us to quantitatively describe the presence of higher-order correlations.

**Figure 1:**
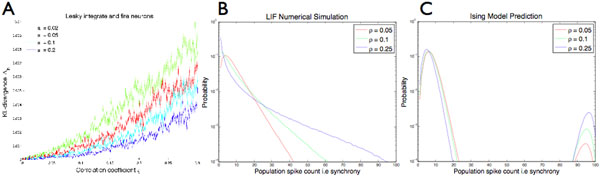
**A**, KL-divergence grows with increasing correlation between the neurons. **B**, Distribution of spike outputs from numerical simulation of LIF neurons. **C**, Predicted distribution of spike outputs from Ising model.

We observe from numerical simulations that for low firing rates, the Kullbach-Leibler divergence grows with increasing correlation i.e. strength of the common input (Figure 1A). For population size N=100, the Ising model predicts a vastly different distribution of spike outputs (Figures 1B,C).

For a leaky IF or exponential IF neuron receiving an input signal identical in all trials, and a background noise independent from trial to trial, it is possible to explicitly calculate the linear response function [2,3]. We use this linear filter to compute instantaneous firing probabilities for the N cells in our setup. This gives us a theoretical basis for our central finding that strong higher-order correlations arise naturally in integrate and fire cells receiving common inputs.
